# Diagnosing cardiovascular disease in western lowland gorillas (*Gorilla gorilla gorilla*) with brain natriuretic peptide

**DOI:** 10.1371/journal.pone.0214101

**Published:** 2019-03-19

**Authors:** Suzan Murray, Jennifer C. Kishbaugh, Lee-Ann C. Hayek, Ilana Kutinsky, Patricia M. Dennis, William Devlin, Katharine L. Hope, Marietta D. Danforth, Hayley W. Murphy

**Affiliations:** 1 Global Health Program, Smithsonian Conservation Biology Institute, National Zoological Park, Washington DC, United States of America; 2 BodeVet, Inc., Rockville, Maryland, United States of America; 3 National Museum of Natural History, Smithsonian Institution, Washington DC, United States of America; 4 Department of Cardiovascular Medicine, William Beaumont Hospital, Royal Oak, Michigan, United States of America; 5 Department of Veterinary Preventive Medicine, Ohio State University, Columbus, Ohio, United States of America; 6 Cleveland Metroparks Zoo, Cleveland, Ohio, United States of America; 7 Oakland University William Beaumont School of Medicine, Rochester, Michigan, United States of America; 8 Department of Animal Health, Smithsonian National Zoological Park, Washington DC, United States of America; 9 Great Ape Heart Project, Zoo Atlanta, Atlanta, Georgia, United States of America; 10 Animal Divisions, Zoo Atlanta, Atlanta, Georgia, United States of America; Scuola Superiore Sant’Anna, ITALY

## Abstract

Cardiovascular disease is a leading cause of death in zoo-housed great apes, accounting for 41% of adult gorilla death in North American zoological institutions. Obtaining a timely and accurate diagnosis of cardiovascular disease in gorillas is challenging, relying on echocardiography which generally requires anesthetic medications that may confound findings and can cause severe side effects in cardiovascularly compromised animals. The measurement of brain natriuretic peptide (BNP) has emerged as a modality of interest in the diagnosis, prognosis and treatment of human patients with heart failure. This study evaluated records for 116 zoo-housed gorillas to determine relationships of BNP with cardiovascular disease. Elevations of BNP levels correlated with the presence of visible echocardiographic abnormalities, as well as reported clinical signs in affected gorillas. Levels of BNP greater 150 pb/mL should alert the clinician to the presence of myocardial strain and volume overload, warranting medical evaluation and intervention.

## Introduction

Cardiovascular disease (CVD) is one of the leading causes of death in both humans and great apes [1–5]. While numerous similarities exist between CVD in these species, there are some unique differences. In the human population, the most common presentation of CVD is congestive heart failure, which is also the leading cause of morbidity, mortality, and hospitalization. This illness is primarily a vascular disease often related to diet and exercise [6]. In contrast, in great apes the most common form of CVD is fibrosing cardiomyopathy of as yet unknown etiology.

Forty-one percent of captive adult western lowland gorilla (*Gorilla gorilla gorilla)* deaths in North American zoological institutions are due to fibrosing cardiomyopathy [7]. In recent decades, great strides have been made in diagnosing and managing great ape CVD. Veterinary medicine has increasingly utilized and adapted diagnostic and therapeutic modalities from human medicine to manage gorilla cardiac disease. Echocardiography has been utilized to create a reference range of normal cardiac measurements, inform diagnostic protocols, and monitor response to treatment in zoologically housed gorillas [8]. However, echocardiography requires specialists to diagnose and interpret findings and often requires anesthesia, which potentially confounds findings [3,8]. This study examines the application of a serum biomarker, brain natriuretic peptide (BNP), as a diagnostic aid for CVD in the zoological gorilla population.

Measurement of serum BNP has emerged as a modality of clinical interest in the diagnosis, prognosis and treatment of human patients with heart failure [6,9]. This neurohormone is secreted by myocardial cells and is particularly associated with the left ventricular myocardial cells in response to cardiomyocyte stretch within the heart [6]. The biomarkers BNP and NT-proBNP (N-terminal pro-brain natriuretic peptide) are the most diagnostic of the natriuretic peptides for cardiac disease [10–12]. In human studies, measuring BNP has been shown to be more sensitive than cardiac ultrasounds for determining early CHF, monitoring patient response to treatment, and determining prognosis [6,9,10,13].

The main utility of BNP in human clinical practice includes having an objective marker of intravascular volume status. Given the morbidity and mortality of CVD in captive gorilla populations, an objective diagnostic tool is needed to allow veterinarians to monitor cardiovascular health, response to treatments, and to aid in determination of when medical intervention is necessary prior to the presence of advanced clinical decompensation. This study examines the usefulness of BNP in diagnosing and predicting cardiac disease in captive gorillas.

## Materials and methods

### Animal selection

Member institutions of the Association of Zoos and Aquariums housing gorillas and participating in the Great Ape Heart Project based at Zoo Atlanta were invited to participate in a population-based cohort study from 2007–2017 examining cardiovascular data following previously reported guidelines for great ape cardiovascular and echocardiographic studies [8]. Standardized data collection sheets were provided requesting patient identification number, patient age, sex, medical history, date of sample acquisition, purpose of procedure, anesthetic medications used for sample acquisition, current medications, and relevant echocardiographic findings. Body weight, if available, was provided. Echocardiographic findings were reviewed by investigators. Post-hoc analysis, where necessary, was performed and measurements were confirmed or calculated off-line by investigators using hand calipers in the case of videotaped studies and on digitally acquired images using Philips R2.5 software (Philips Healthcare, Andover, Massachusetts 01810, USA) and GE Vivid-I (General Electric, Milwaukee, Wisconsin 53209, USA). Cardiac parameters measured included aortic root diameter (Ao Rt), left atrial size (L atrium), and left ventricle (LV) measurements including LV internal diameter in systole (LVID), and diastole (LVIDd), as well as diastolic septal (IVS) and posterior wall thickness (LVPW). For the purpose of data entry, estimated ejection fractions (EF) were given a numerical value that represented the average EF.

Data and BNP samples were collected under anesthesia with regimens varying based on institution. Medications used for anesthesia included ketamine, tiletamine-zolazepam, medetomidine, propofol, isoflurane or sevoflurane inhalant anesthesia, among others with protocols differing between institution and individual animal. Per standard practice among the majority of facilities, gorillas were fasted prior to sedation. Data were entered into Excel (Microsoft, PTSGE Corp., Seattle, Washington 98104, USA) spreadsheets. Whole blood and plasma samples in EDTA taken at the time of evaluation were submitted to the Smithsonian’s National Zoological Park along with the standard data collection sheet. All samples were processed within 48 hours of sampling on the Triage BNP test machine (Triage BNP Test, Biosite, San Diego, CA, USA) according to manufacturer recommendations. The amount of BNP present in the sample were displayed as a number in pg/mL. For the purpose of statistical analysis, readings that read as “< 5” were converted to the value of “4”, and readings that read as “> 5,000” were converted to the value of “5,000”.

### Statistical methods

All descriptive statistics and analyses were run on SPSS V20 and MathCad 2013. Descriptive statistics were calculated for all variables over all gorillas and subgroups of sex and health. Exploratory analyses were examined by grouping but were not useful because subjective groupings based upon medical diagnoses proved more reliable. Assumptions were tested for each test of hypothesis and variance-stabilizing transformations were applied using natural logs and Box-Cox methodology accordingly. When correlations were computed they were Pearson’s for continuous measures and Spearman’s was used to test measures with two or more categories (dichotomous or polychotomous) measures. General linear models were developed using factors (sex, health) as well as covariates (age) and terms for nonlinearity if necessary. Since age was highly correlated with health status, age was used as a covariate in the model to increase precision of the model design. This procedure statistically removes from the model that part of the variability that is predictable from age alone. Canonical discriminant analyses were modeled when required as a natural extension of regression analysis for predicting group membership. Post hoc classification tables were based upon Fisher’s classification functions.

## Results

Records were analyzed for 116 zoo-housed gorillas 10 years of age and over, 51 females and 65 males. Gorillas were assigned into groups based upon health assessment: Group 1 (n = 85) consisted of gorillas that were apparently healthy; Group 2 (n = 9) contained gorillas demonstrating clinical signs consistent with cardiovascular decompensation including but not limited to coughing, lethargy, exercise intolerance, social withdrawal, dyspnea, or grabbing at the chest; and Group 3 (n = 22) contained gorillas that were currently asymptomatic, and were undergoing medical management for CVD based upon diagnosis via previous echocardiographic examination.

**Fig 1:**
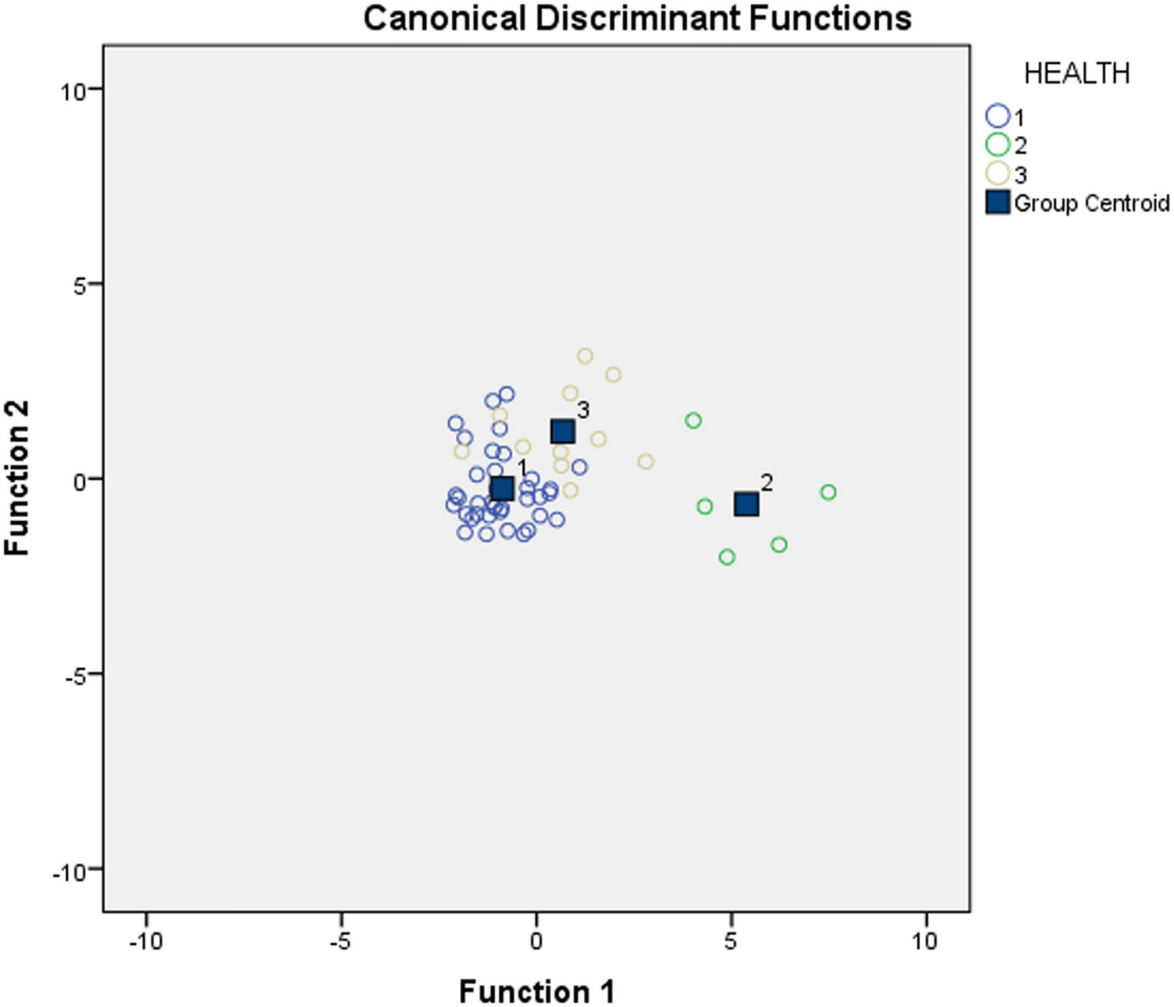
Canonical discriminant functions describing the cluster patterns of brain natriuretic peptide results for Group 1 (healthy), Group 2 (clinically ill), and Group 3 (newly diagnosed) zoo-housed gorillas. The three groups are significantly different as demonstrated by cluster patterns.

Results of BNP testing from individual animals in these groups were examined through canonical discriminant functions (Fig 1). The means of the assigned groups appeared to discriminate in to clearly clusters demonstrating that the three groups were statistically distinct; Group 1 demonstrated low value clustering, Group 2 high value clustering, and Group 3 clustering in the middle though towards a lower range. The descriptive values for Age, BNP value, and Cardiac Measurements for Groups 1, 2 and 3 are listed in Table 1.

**Table 1:** Age, BNP level, diastolic interventricular septal wall thickness (IVSd), left ventricular internal diameter in diastole (LVIDd), left ventricular diastolic posterior wall thickness (LVPWd), and ejection fraction (EF) measurements for Groups 1, 2 and 3.

		GROUP 1	GROUP 2	GROUP 3
**AGE (years)**	**N**	85	9	22
	**Mean ± SD**	19.85 ± 8.410	35.56 ± 11.092	28.32 ± 9.357
	**Median**	17	39	28
	**Range**	18–72	21–49	14–48
**BNP (pg/mL)**	**N**	85	9	22
	**Mean ± SD**	23.51 ± 21.63	2785.11 ± 2042.595	65.92 ± 61.137
	**Median**	16.2	2000	46.65
	**Range**	4–130	259–5000	4–240
**IVSD (cm)**	**N**	42	6	14
	**Mean ± SD**	1.23 ± 0.378	1.77 ± 0.525	1.78 ± 0.446
	**Median**	1.22	1.65	1.7
	**Range**	0.70–2.69	1.30–2.39	1.11–2.70
**LVIDd (cm)**	**N**	43	5	14
	**Mean ± SD**	4.69 ± 0.84	7.03 ± 2.648	5.34 ± 1.140
	**Median**	4.49	7.28	5.35
	**Range**	2.90–6.40	3.50–10.80	3.15–7.40
**LVPWd (cm)**	**N**	41	5	13
	**Mean ± SD**	1.24 ± 0.389	1.79 ± 0.502	1.78 ± 0.452
	**Median**	1.17	1.8	1.7
	**Range**	0.70–2.41	1.20–2.54	1.18–2.60
**EF (%)**	**N**	41	6	11
	**Mean ± SD**	64.83 ± 8.209	30.50 ± 14.039	54.64 ± 18.057
	**Median**	65	35	55
	**Range**	50–82	16–81	20–80

Correlations of BNP and cardiac measurements were significant and positive for age (r = 0.283, p = 0.002) and LVIDd (r = 0.569, p = 0.0001), while the correlation of EF and BNP was significant and negative (r = -0.667, n = 58, p<0.0001). Levene’s test by sex (SD_female_ = 1.489, SD_male_ = 1.642) was not significant (p = 0.473). A pooled variance t-test was used to examine the difference between male (3.632) and female (2.910) lnBNP means in each group and was significant (p = 0.016). Male BNP values showed significant positive correlation with age (r = 0.1262, n = 65, p = 0.035) and LVIDd (r = 0.679, n = 37, p = 00001), and negative correlation with EF (r = -0.681, n = 34, p<0.00001) but not health status (r = 0.094, n = 65, p = 0.455 ns). Female BNP values showed no correlation with LVIDd (r = -0.226, n = 25, p = 0.276 ns) but significant negative correlation with EF (r = -0.529, n = 24, p = 0.008) and positive correlation with age (r = 0.345, n = 51, p = 0.013) and health status (r = 0.484, n = 51, p = 0.0003). When examined by health status the Group 1 BNP results did not correlate with LVIDd, EF or with age. Group 2 BNP results likewise had no correlates. Group 3 correlated BNP values with age (r = 0.470, n = 22, p = 0.027) and LVPWd (r = 0.570, n = 13, p = 0.042).

General linear models were examined using sex and health status as factors with their interaction and age, which was highly significant across health status, was used as a covariate. Analysis of covariates provides for an age ‘adjustment’ across the groups and thereby can increase precision of the model. In each case worsening health status was significantly correlated to increased BNP values at p<0.001 level. To discriminate health status using BNP with echocardiographic variables, the best predictive model used utilized BNP, EF and LVPWd with an 80% jackknifed classification rate. With age added, an overall 87% correct, though biased, classification was obtained, with 90%, 100% and 75% correct in Groups 1, 2, and 3, respectively.

## Discussion

Elevation of BNP levels correlates with the presence of visible echocardiographic abnormalities, as well as reported clinical signs. Given the BNP ranges present within each assigned health group it was found that BNP levels from <5.0–70 pg/dL were related to animals without evidence of CDV, BNP levels of 10–200 pg/dL were associated with animals demonstrating echocardiographic evidence of CVD that were currently being managed by medication, and BNP levels greater than 200 pg/dL were generally found in animals displaying reported clinical signs and echocardiographic evidence of decompensating CVD. Analysis of the data demonstrated that BNP levels increased with worsening cardiovascular health status, including increased IVSd, LVIDd, and LVPWd, as well as with increasing age; BNP values also increased with a decline in EF and cardiac function. Sex was not found to be linked to differences in BNP measurements, contrary to studies in human medicine [11]. It was also noted that while animals receiving medications to manage CVD displayed lower levels of BNP, the animals still displayed echocardiographic changes consistent with CVD. These data mirror reports in human medicine, in which clinical signs are improved and BNP levels are lower in patients under current medical treatment for cardiac disease as compared to newly diagnosed and as yet untreated patients [14]. Interestingly, animals with BNP levels measuring greater than 1000 pg/mL were all deceased within 6 months of sample analysis. To fully understand the varying levels of BNP in various stages of cardiac disease, longitudinal studies of individuals would need to be evaluated.

One gorilla not included in analysis with a history of severe renal dysfunction without clinical CVD had a dramatic elevation in BNP levels, which may correlate to the finding that increased intravascular volume results in increased secretion of BNP as a result of either cardiac decompensation or renal dysfunction [13]. While animals may present with nonspecific clinical signs of illness and an elevated BNP level, this emphasizes the need for additional diagnostic testing. The clinical signs noted at the presentation of ill animals (lethargy, shortness of breath, pressing on the thoracic area with the palms or fingers, anorexia, weight loss, coughing, gastroesophageal reflux or regurgitation), while not pathognomonic for cardiac disease, have many similarities with clinical signs reported by human patients presenting with cardiac disease. Although this study did not aim to evaluate or classify signs of illness, this provides a useful guide to the veterinary clinician.

Levels of BNP should be taken into consideration with echocardiograph examination, electrocardiograms, dietary evaluation, blood parameters, radiography, and environmental settings to obtain a complete picture of cardiovascular health, disease severity and progression, allowing for treatment modifications [4]. Our data demonstrates that a BNP level over 150 pb/mL in a gorilla with normal renal function should alert the clinician that there is significant myocardial strain and volume overload, warranting medical evaluation and intervention.

## Supporting information

S1 Fig BNP for all study gorillas. Brain natriuretic peptide (pg/ml) histogram plots with log transformation applied for a) all gorillas included in the study (n = 116); b) gorillas assigned a health status of “1” (n = 85); c) gorillas assigned a health status of “2” (n = 9); and d) gorillas assigned a health status of “3” (n = 22).
(ZIP)

S2 Fig Age and echocardiographic findings for gorillas with health status “1”. Histogram plots with normal curves applied depicting for all gorillas: a) age (years); b) interventricular septal end diastole (IVSd; cm); c) left ventricular internal diameter end diastole (LVIDd; cm); d) left ventricular posterior wall diastole (LVPWd; cm); and e) ejection fraction (EF; %).
(ZIP)

S3 Fig Age and echocardiographic findings for gorillas with health status “1”. Histogram plots with normal curves applied depicting gorillas assigned a health status of “1”: a) age (years); b) interventricular septal end diastole (IVSd; cm); c) left ventricular internal diameter end diastole (LVIDd; cm); d) left ventricular posterior wall diastole (LVPWd; cm); and e) ejection fraction (EF; %).
(ZIP)

S4 Fig Age and echocardiographic findings for gorillas with health status “2”. Histogram plots with normal curves applied depicting gorillas assigned a health status of “2”: a) age (years); b) interventricular septal end diastole–IVSd (cm); c) left ventricular internal diameter end diastole–LVIDd (cm); d) left ventricular posterior wall diastole–LVPWd (cm); and e) ejection fraction–EF (%).
(ZIP)

S5 Fig Age and echocardiographic findings for gorillas with health status “3”. Histogram plots with normal curves applied depicting gorillas assigned a health status of “3”: a) age (years); b) interventricular septal end diastole–IVSd (cm); c) left ventricular internal diameter end diastole–LVIDd (cm); d) left ventricular posterior wall diastole–LVPWd (cm); and e) ejection fraction–EF (%).
(ZIP)

S1 Table Raw data used for statistical evaluation of all gorillas. (XLSX)

## References

[1] Lowenstine LJ, McManamon R, Terio KA. Comparative Pathology of Aging Great Apes: Bonobos, Chimpanzees, Gorillas, and Orangutans. Vet Pathol. 2016 3;53(2):250–76. 10.1177/0300985815612154 26721908

[2] Gamble KC, North MCK, Backues K, Ross SR. Pathologic review of the chimpanzee (Pan troglodytes): 1990–2003. In: Annual Conference of the American Association of Zoo Veterinarians. 2004. p. 561–566.

[3] McManamon R, Lowenstine L. Cardiovascular Disease in Great Apes In: Fowler’s Zoo and Wild Animal Medicine [Internet]. Elsevier; 2012 [cited 2018 Nov 9]. p. 408–15. http://linkinghub.elsevier.com/retrieve/pii/B9781437719864000536

[4] Rush EM, Ogburn AL, Monroe D. Clinical Management of a Western Lowland Gorilla (*Gorilla gorilla gorilla*) with a Cardiac Resynchronization Therapy Device. J Zoo Wildl Med. 2011 6;42(2):263–76. 10.1638/2009-0080.1 22946404

[5] Sidney S, Quesenberry CP, Jaffe MG, Sorel M, Nguyen-Huynh MN, Kushi LH, et al Recent Trends in Cardiovascular Mortality in the United States and Public Health Goals. JAMA Cardiol. 2016 8 1;1(5):594 10.1001/jamacardio.2016.1326 27438477

[6] Raizada A, Bhandari S, Khan MA, Singh HV, Thomas S, Sarabhai V, et al Brain type natriuretic peptide (BNP)—A marker of new millennium in diagnosis of congestive heart failure. Indian J Clin Biochem. 2007 3;22(1):4–9. 10.1007/BF02912873 23105644 PMC3454260

[7] Schulman FY, Farb A, Virmani R, Montali RJ. Fibrosing Cardiomyopathy in Captive Western Lowland Gorillas (Gorilla gorilla gorilla) in the United States: A Retrospective Study. J Zoo Wildl Med. 1995;26(1):43–51.

[8] Murphy HW, Dennis P, Devlin W, Meehan T, Kutinsky I. Echocardiographic Parameters of Captive Western Lowland Gorillas (*Gorilla gorilla gorilla*). J Zoo Wildl Med. 2011 12;42(4):572–9. 10.1638/2010-0139.1 22204050

[9] Bettencourt P. Brain Natriuretic Peptide (Nesiritide) in the Treatment of Heart Failure. Cardiovasc Drug Rev. 2006 6 7;20(1):27–36. 10.1111/j.1527-3466.2002.tb00080.x 12070532

[10] Hunt PJ, Richards AM, Nicholls MG, Yandle TG, Doughty RN, Espiner EA. Immunoreactive amino-terminal pro-brain natriuretic peptide (NT-PROBNP): a new marker of cardiac impairment. Clin Endocrinol (Oxf). 1997 9;47(3):287–96. 9373449 10.1046/j.1365-2265.1997.2361058.x

[11] Maisel AS, Clopton P, Krishnaswamy P, Nowak RM, McCord J, Hollander JE, et al Impact of age, race, and sex on the ability of B-type natriuretic peptide to aid in the emergency diagnosis of heart failure: results from the Breathing Not Properly (BNP) multinational study. Am Heart J. 2004 6;147(6):1078–84. 10.1016/j.ahj.2004.01.013 15199359

[12] Srisawasdi P, Vanavanan S, Charoenpanichkit C, Kroll MH. The Effect of Renal Dysfunction on BNP, NT-proBNP, and Their Ratio. Am J Clin Pathol. 2010 1;133(1):14–23. 10.1309/AJCP60HTPGIGFCNK 20023254

[13] Maeda K, Tsutamoto T, Wada A, Hisanaga T, Kinoshita M. Plasma brain natriuretic peptide as a biochemical marker of high left ventricular end-diastolic pressure in patients with symptomatic left ventricular dysfunction. Am Heart J. 1998 5;135(5):825–32. 9588412 10.1016/s0002-8703(98)70041-9

[14] Dhaliwal AS, Deswal A, Pritchett A, Aguilar D, Kar B, Souchek J, et al Reduction in BNP Levels With Treatment of Decompensated Heart Failure and Future Clinical Events. J Card Fail. 2009 5;15(4):293–9. 10.1016/j.cardfail.2008.11.007 19398076

