# Supplementary figures and images for: Diagnosing cardiovascular disease in western lowland gorillas (Gorilla gorilla gorilla) with brain natriuretic peptide
Source: PLoS One. 2019 Mar 19;14(3):e0214101. doi: 10.1371/journal.pone.0214101 (PMC6424555; doi:10.1371/journal.pone.0214101)

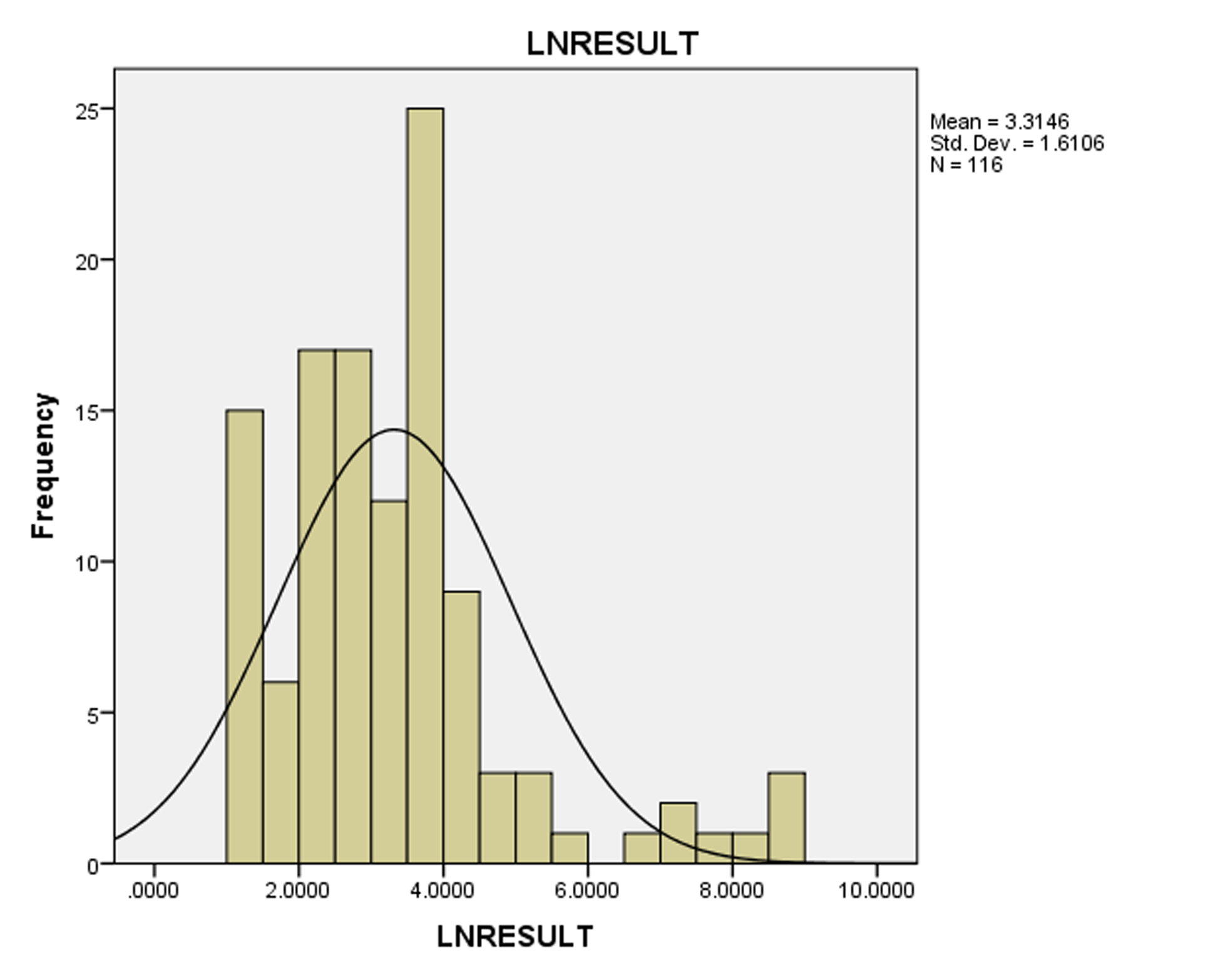

Supplement: S1 Fig — Brain natriuretic peptide (pg/ml) histogram plots with log transformation applied for a) all gorillas included in the study (n = 116); b) gorillas assigned a health status of “1” (n = 85); c) gorillas assigned a health status of “2” (n = 9); and d) gorillas assigned a health status of “3” (n = 22). (ZIP) [file pone.0214101.s001.zip › S1A.tif]

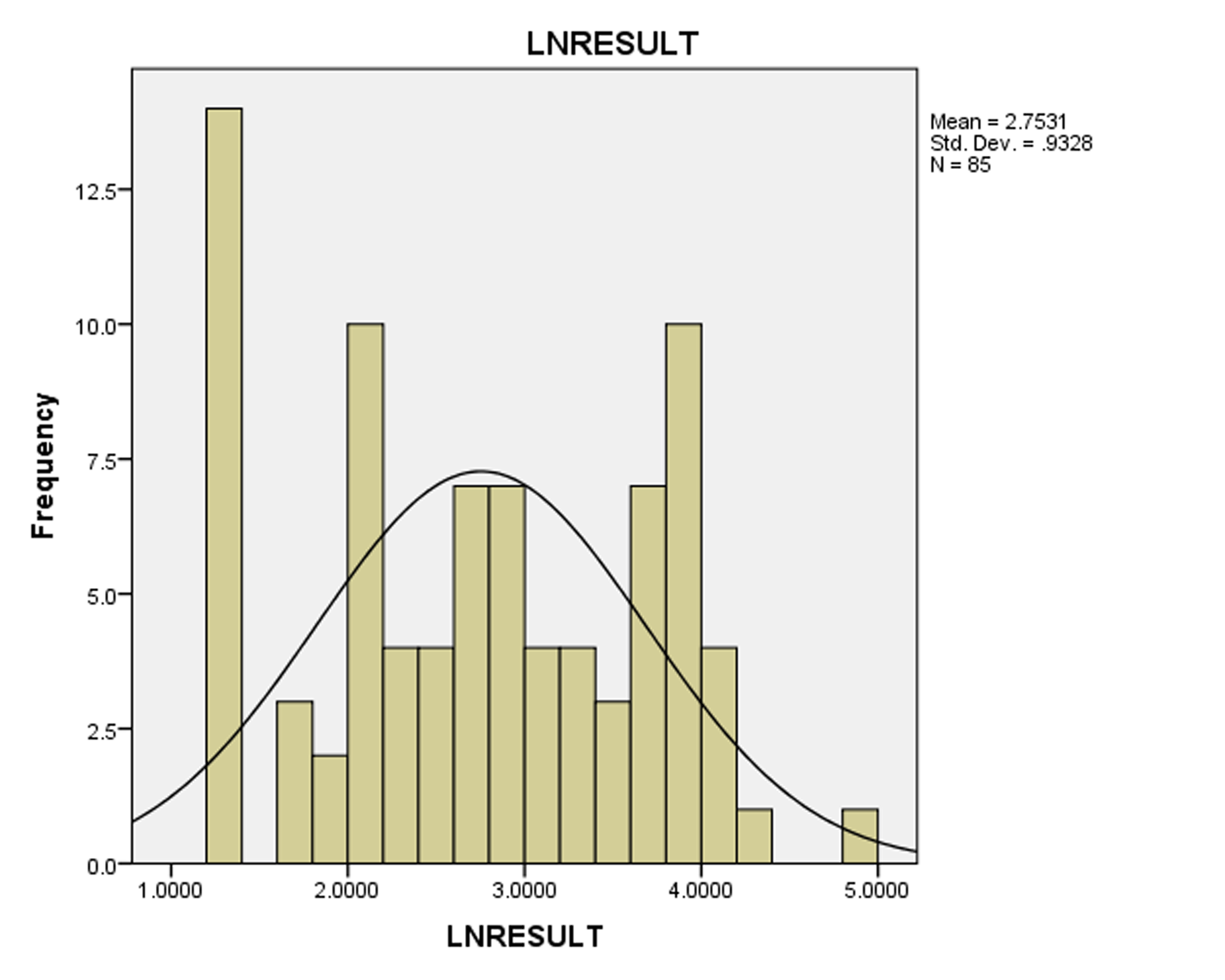

Supplement: S1 Fig — Brain natriuretic peptide (pg/ml) histogram plots with log transformation applied for a) all gorillas included in the study (n = 116); b) gorillas assigned a health status of “1” (n = 85); c) gorillas assigned a health status of “2” (n = 9); and d) gorillas assigned a health status of “3” (n = 22). (ZIP) [file pone.0214101.s001.zip › S1B.tif]

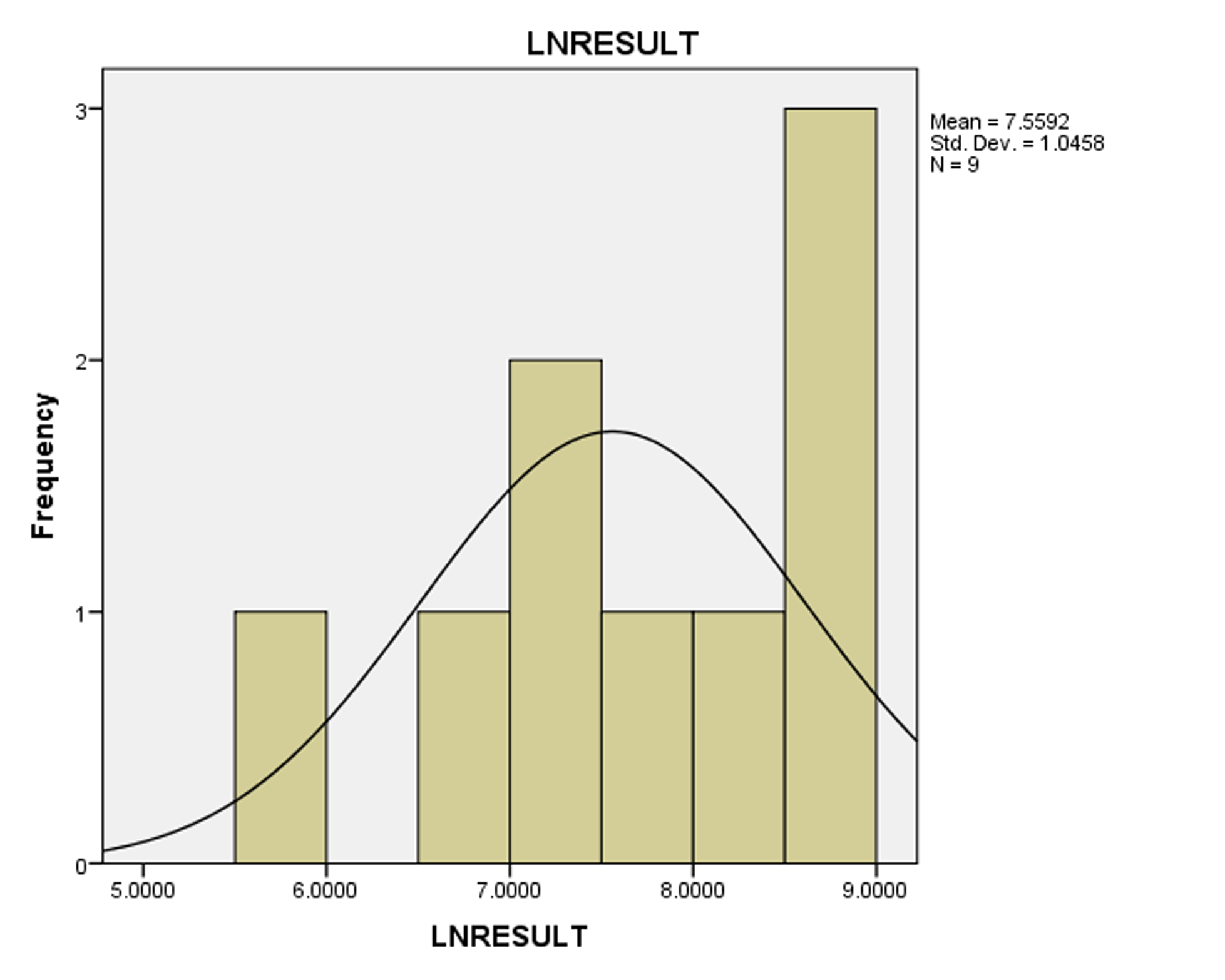

Supplement: S1 Fig — Brain natriuretic peptide (pg/ml) histogram plots with log transformation applied for a) all gorillas included in the study (n = 116); b) gorillas assigned a health status of “1” (n = 85); c) gorillas assigned a health status of “2” (n = 9); and d) gorillas assigned a health status of “3” (n = 22). (ZIP) [file pone.0214101.s001.zip › S1C.tif]

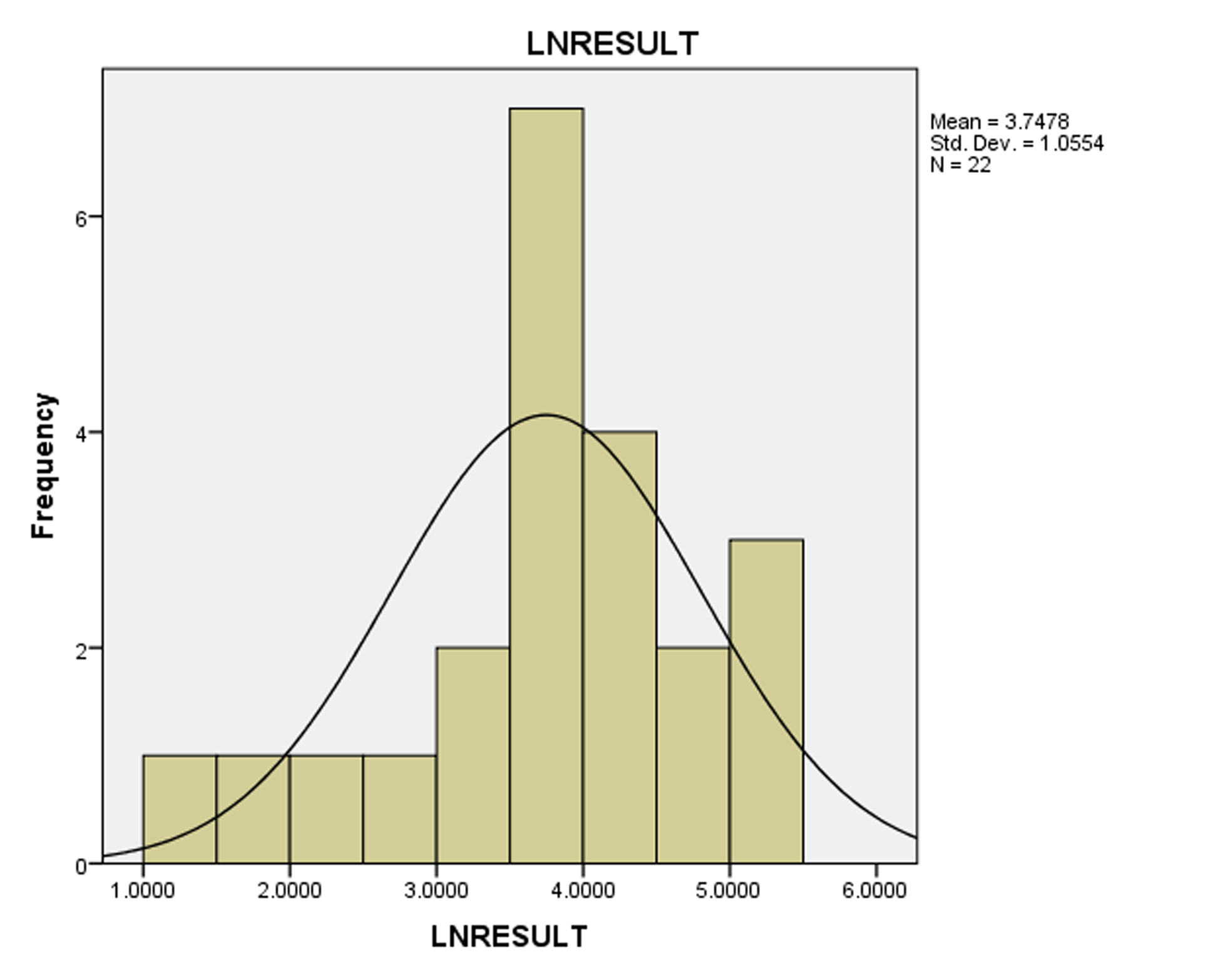

Supplement: S1 Fig — Brain natriuretic peptide (pg/ml) histogram plots with log transformation applied for a) all gorillas included in the study (n = 116); b) gorillas assigned a health status of “1” (n = 85); c) gorillas assigned a health status of “2” (n = 9); and d) gorillas assigned a health status of “3” (n = 22). (ZIP) [file pone.0214101.s001.zip › S1D.tif]

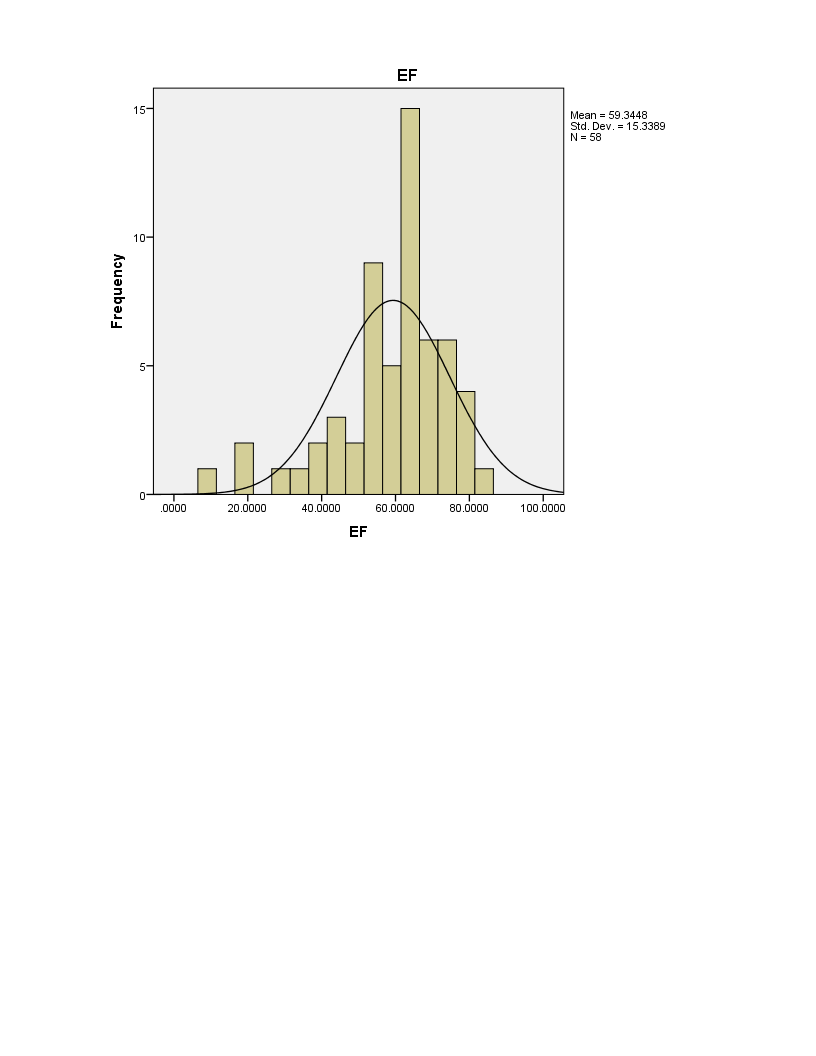

Supplement: S2 Fig — Histogram plots with normal curves applied depicting for all gorillas: a) age (years); b) interventricular septal end diastole (IVSd; cm); c) left ventricular internal diameter end diastole (LVIDd; cm); d) left ventricular posterior wall diastole (LVPWd; cm); and e) ejection fraction (EF; %). (ZIP) [file pone.0214101.s002.zip › S2E.tif]

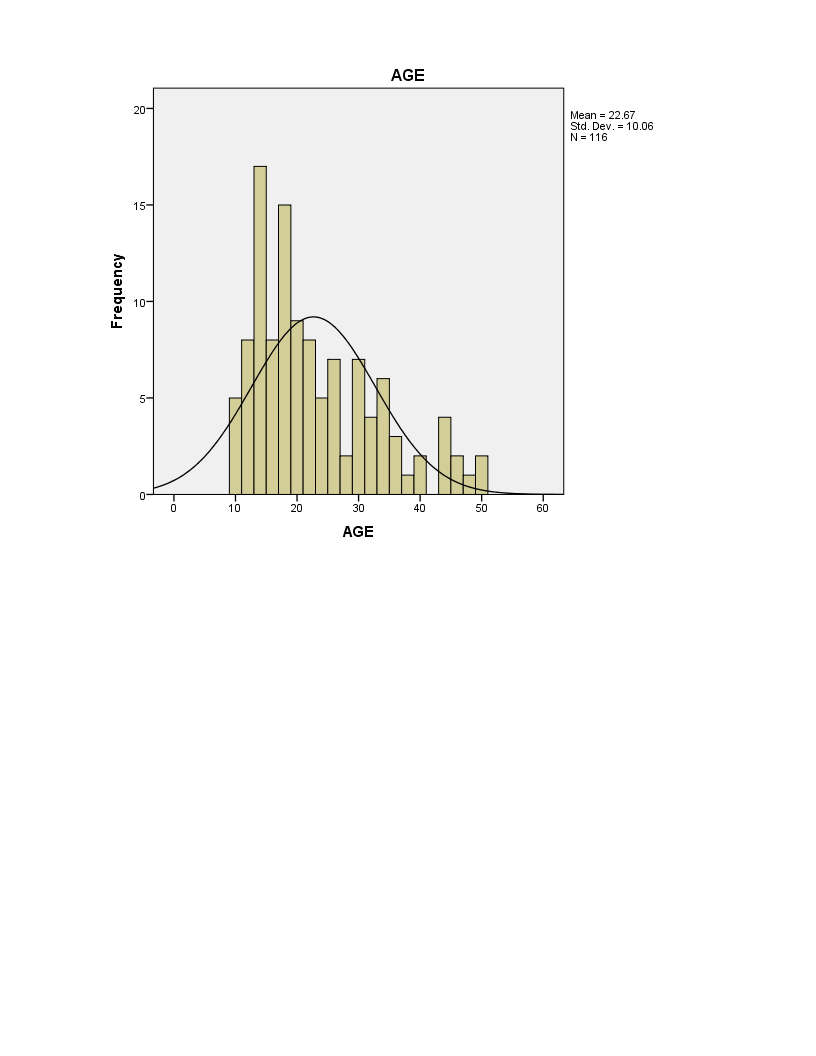

Supplement: S2 Fig — Histogram plots with normal curves applied depicting for all gorillas: a) age (years); b) interventricular septal end diastole (IVSd; cm); c) left ventricular internal diameter end diastole (LVIDd; cm); d) left ventricular posterior wall diastole (LVPWd; cm); and e) ejection fraction (EF; %). (ZIP) [file pone.0214101.s002.zip › S2A.tif]

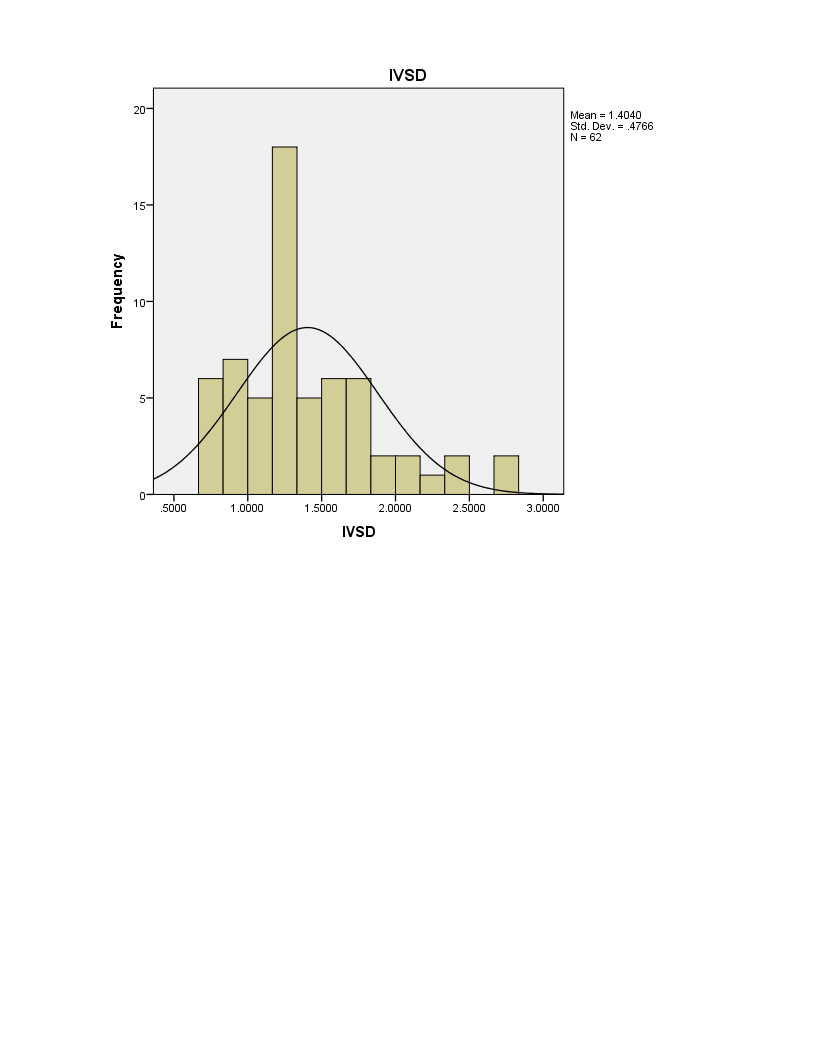

Supplement: S2 Fig — Histogram plots with normal curves applied depicting for all gorillas: a) age (years); b) interventricular septal end diastole (IVSd; cm); c) left ventricular internal diameter end diastole (LVIDd; cm); d) left ventricular posterior wall diastole (LVPWd; cm); and e) ejection fraction (EF; %). (ZIP) [file pone.0214101.s002.zip › S2B.tif]

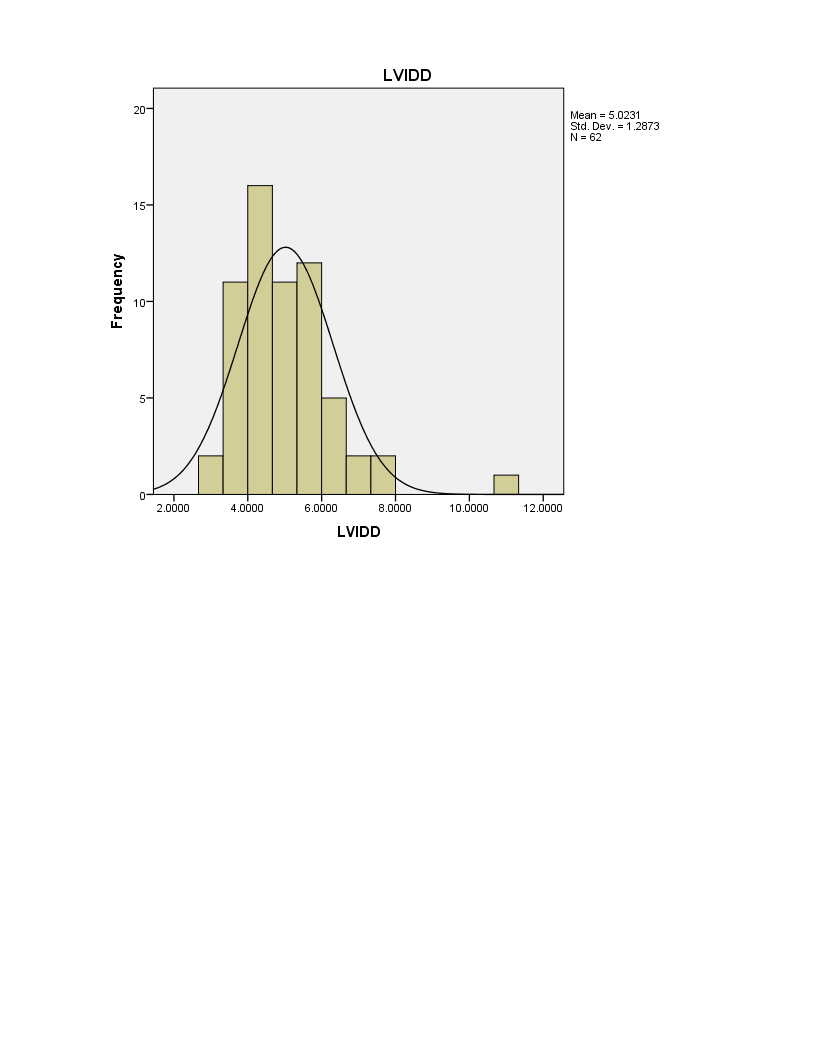

Supplement: S2 Fig — Histogram plots with normal curves applied depicting for all gorillas: a) age (years); b) interventricular septal end diastole (IVSd; cm); c) left ventricular internal diameter end diastole (LVIDd; cm); d) left ventricular posterior wall diastole (LVPWd; cm); and e) ejection fraction (EF; %). (ZIP) [file pone.0214101.s002.zip › S2C.tif]

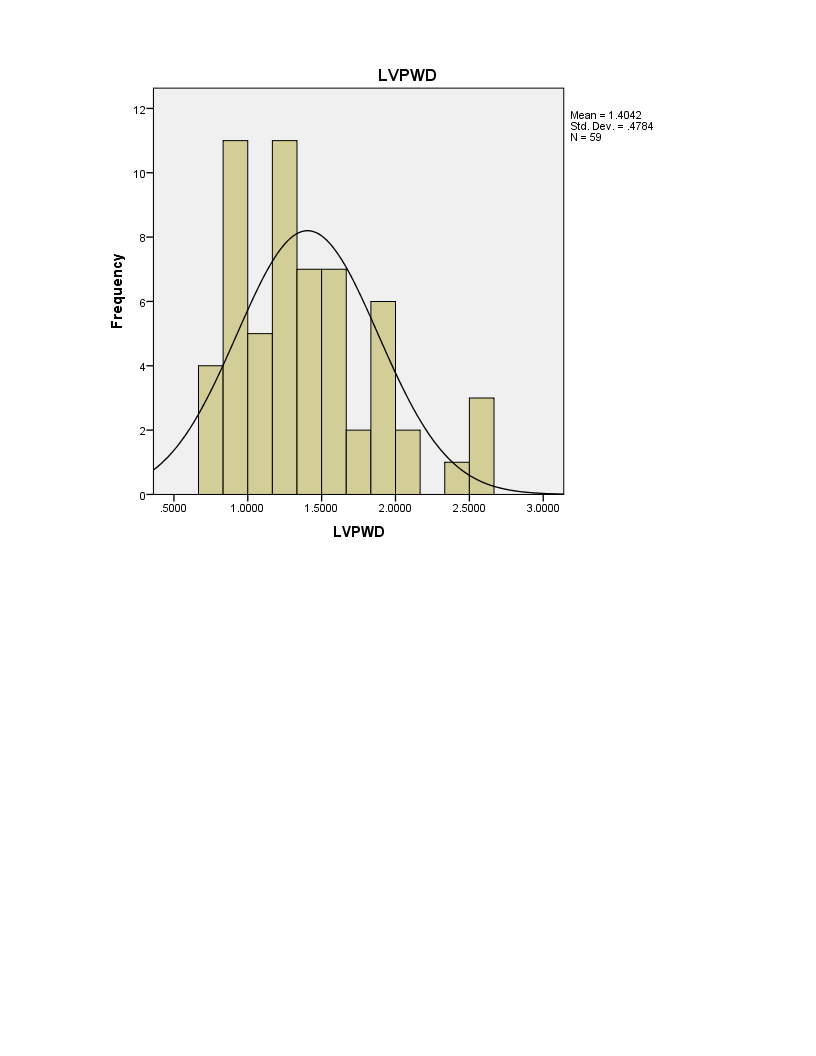

Supplement: S2 Fig — Histogram plots with normal curves applied depicting for all gorillas: a) age (years); b) interventricular septal end diastole (IVSd; cm); c) left ventricular internal diameter end diastole (LVIDd; cm); d) left ventricular posterior wall diastole (LVPWd; cm); and e) ejection fraction (EF; %). (ZIP) [file pone.0214101.s002.zip › S2D.tif]

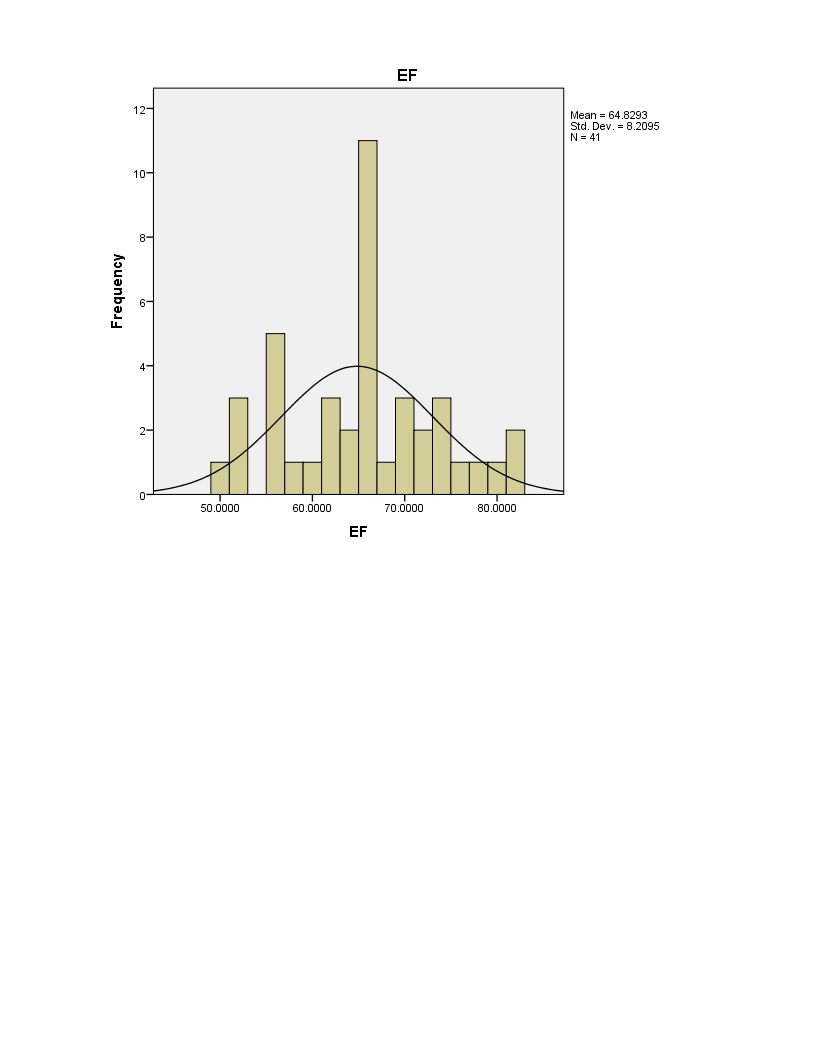

Supplement: S3 Fig — Histogram plots with normal curves applied depicting gorillas assigned a health status of “1”: a) age (years); b) interventricular septal end diastole (IVSd; cm); c) left ventricular internal diameter end diastole (LVIDd; cm); d) left ventricular posterior wall diastole (LVPWd; cm); and e) ejection fraction (EF; %). (ZIP) [file pone.0214101.s003.zip › S3E.tif]

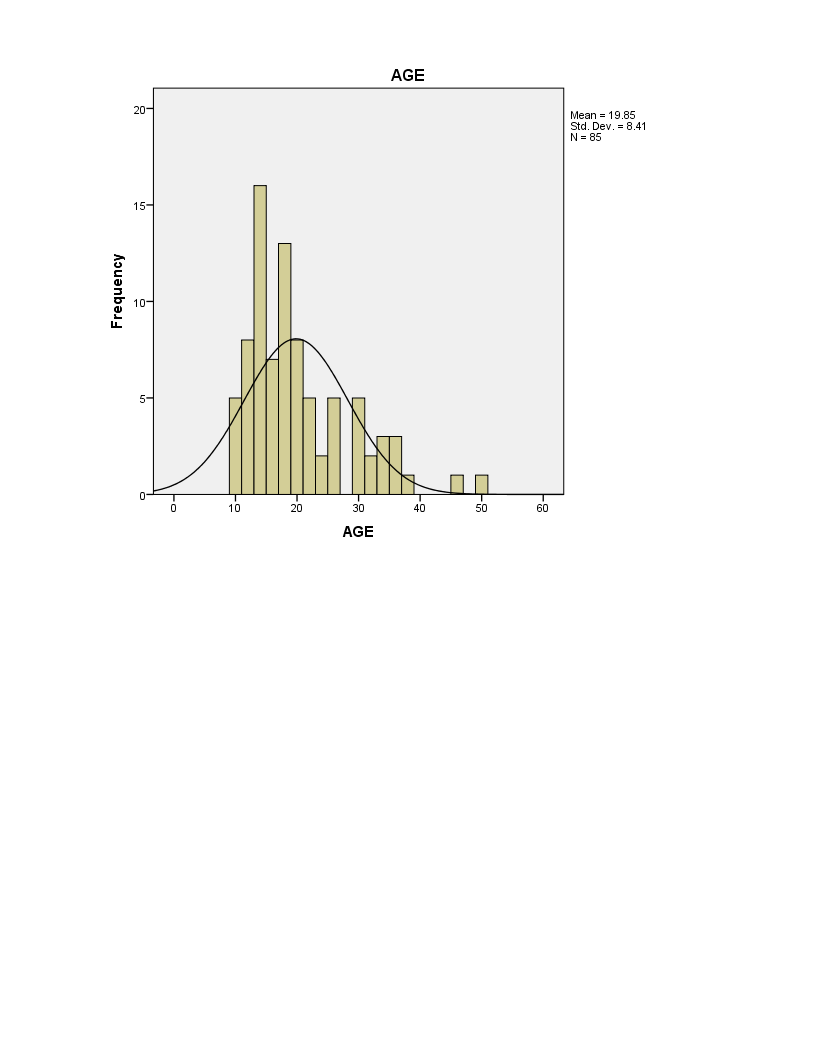

Supplement: S3 Fig — Histogram plots with normal curves applied depicting gorillas assigned a health status of “1”: a) age (years); b) interventricular septal end diastole (IVSd; cm); c) left ventricular internal diameter end diastole (LVIDd; cm); d) left ventricular posterior wall diastole (LVPWd; cm); and e) ejection fraction (EF; %). (ZIP) [file pone.0214101.s003.zip › S3A.tif]

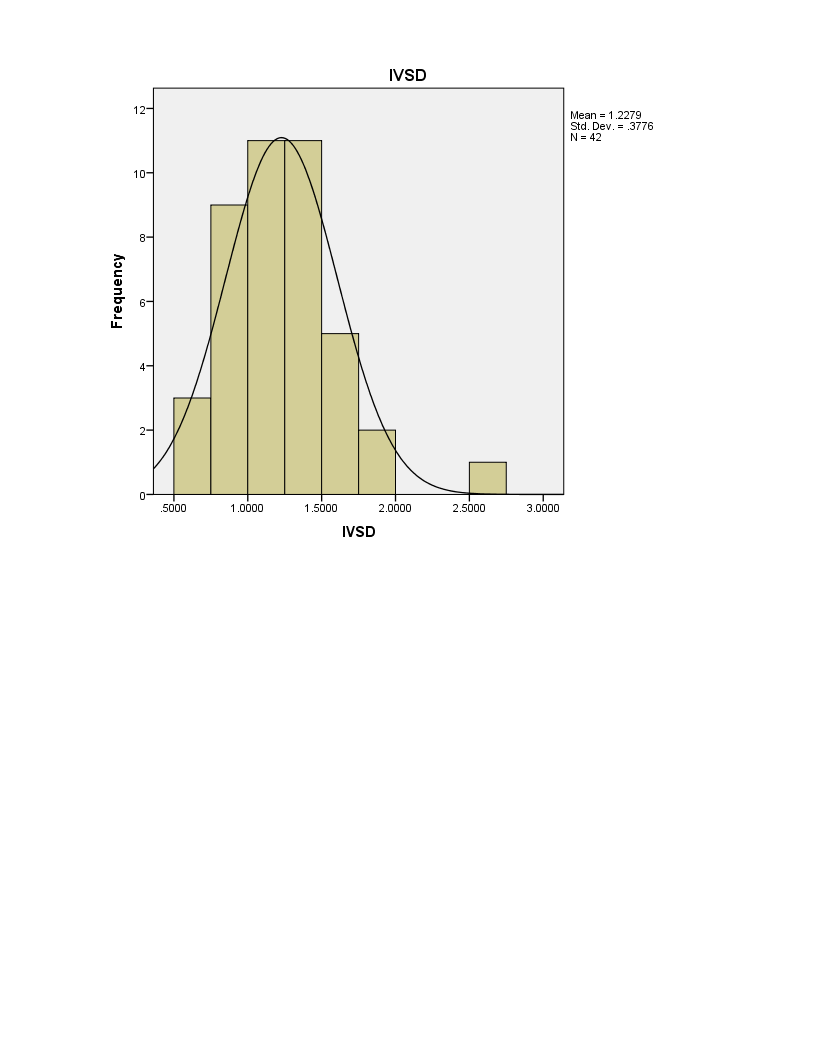

Supplement: S3 Fig — Histogram plots with normal curves applied depicting gorillas assigned a health status of “1”: a) age (years); b) interventricular septal end diastole (IVSd; cm); c) left ventricular internal diameter end diastole (LVIDd; cm); d) left ventricular posterior wall diastole (LVPWd; cm); and e) ejection fraction (EF; %). (ZIP) [file pone.0214101.s003.zip › S3B.tif]

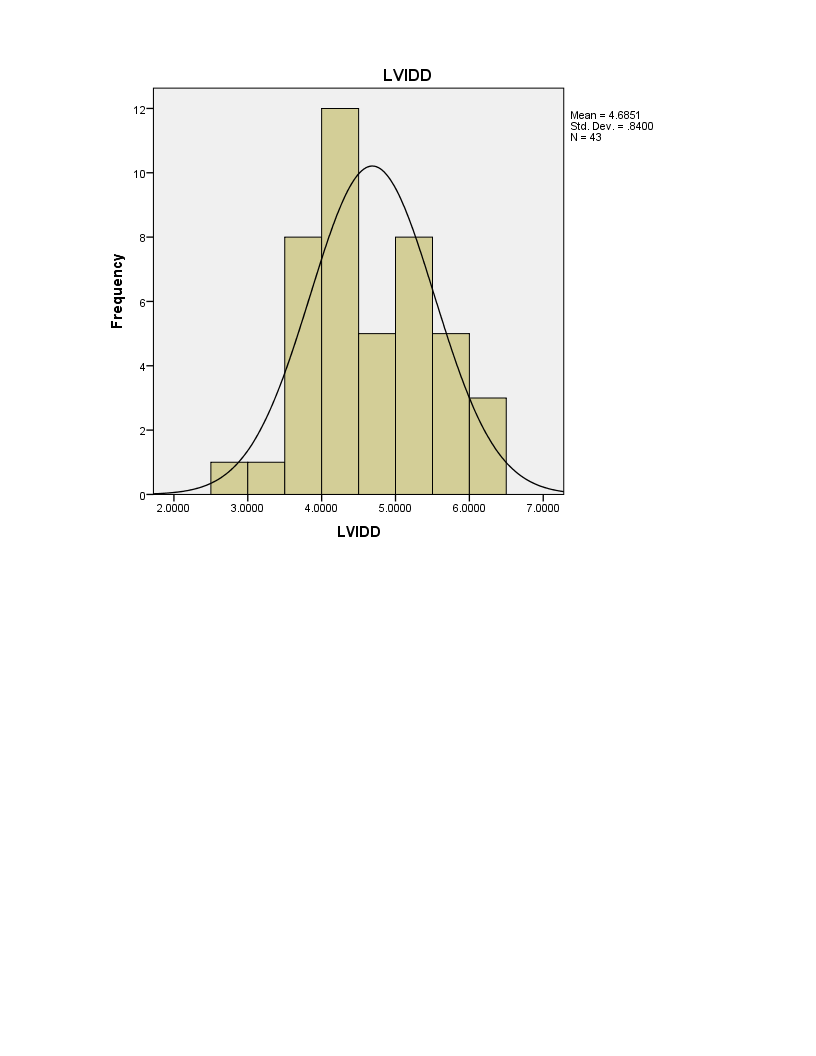

Supplement: S3 Fig — Histogram plots with normal curves applied depicting gorillas assigned a health status of “1”: a) age (years); b) interventricular septal end diastole (IVSd; cm); c) left ventricular internal diameter end diastole (LVIDd; cm); d) left ventricular posterior wall diastole (LVPWd; cm); and e) ejection fraction (EF; %). (ZIP) [file pone.0214101.s003.zip › S3C.tif]

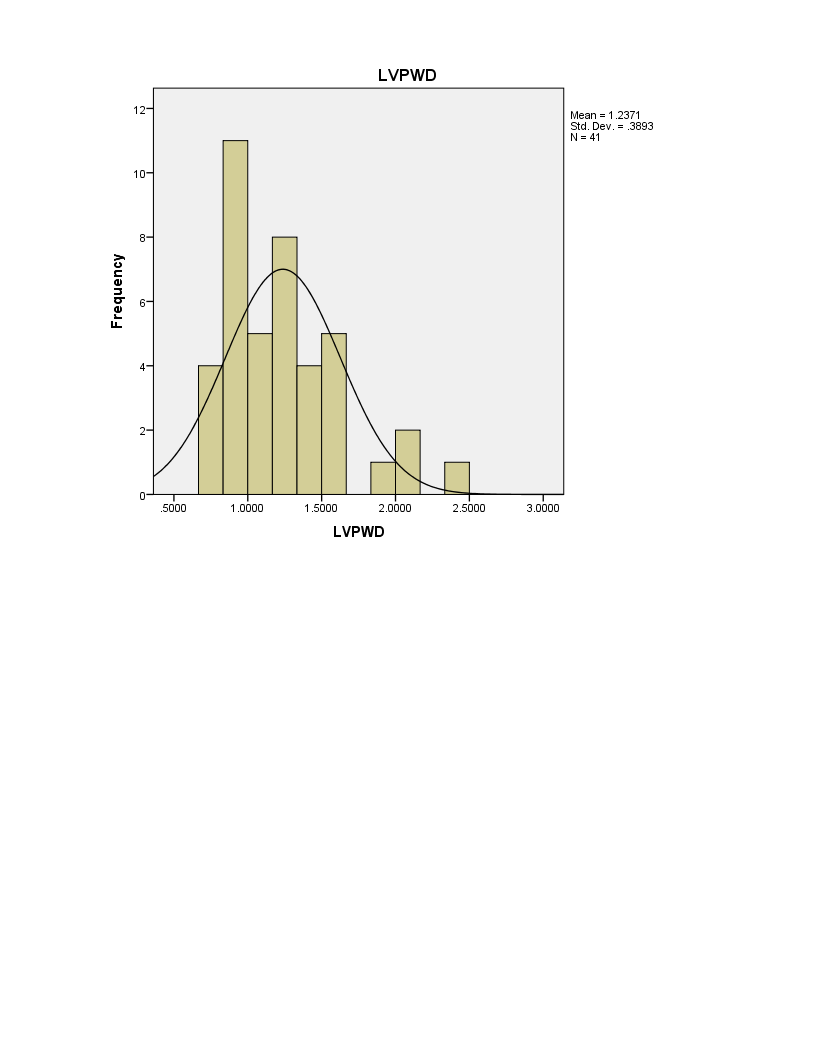

Supplement: S3 Fig — Histogram plots with normal curves applied depicting gorillas assigned a health status of “1”: a) age (years); b) interventricular septal end diastole (IVSd; cm); c) left ventricular internal diameter end diastole (LVIDd; cm); d) left ventricular posterior wall diastole (LVPWd; cm); and e) ejection fraction (EF; %). (ZIP) [file pone.0214101.s003.zip › S3D.tif]

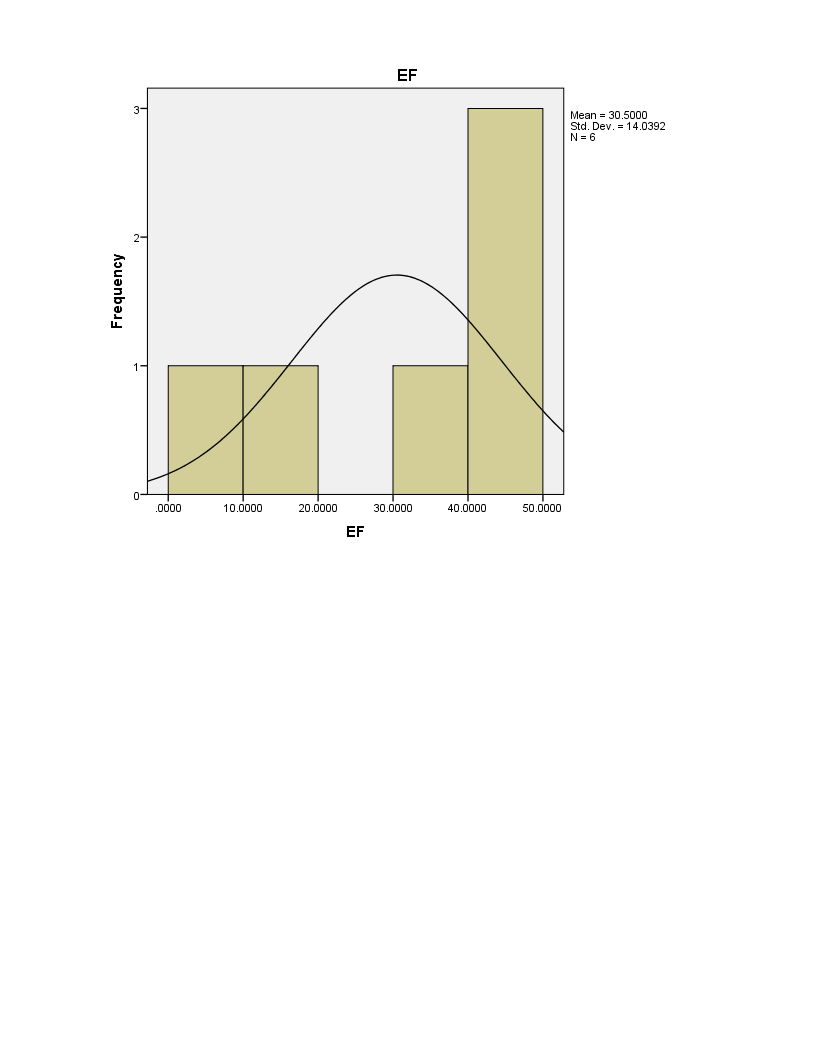

Supplement: S4 Fig — Histogram plots with normal curves applied depicting gorillas assigned a health status of “2”: a) age (years); b) interventricular septal end diastole–IVSd (cm); c) left ventricular internal diameter end diastole–LVIDd (cm); d) left ventricular posterior wall diastole–LVPWd (cm); and e) ejection fraction–EF (%). (ZIP) [file pone.0214101.s004.zip › S4E.tif]

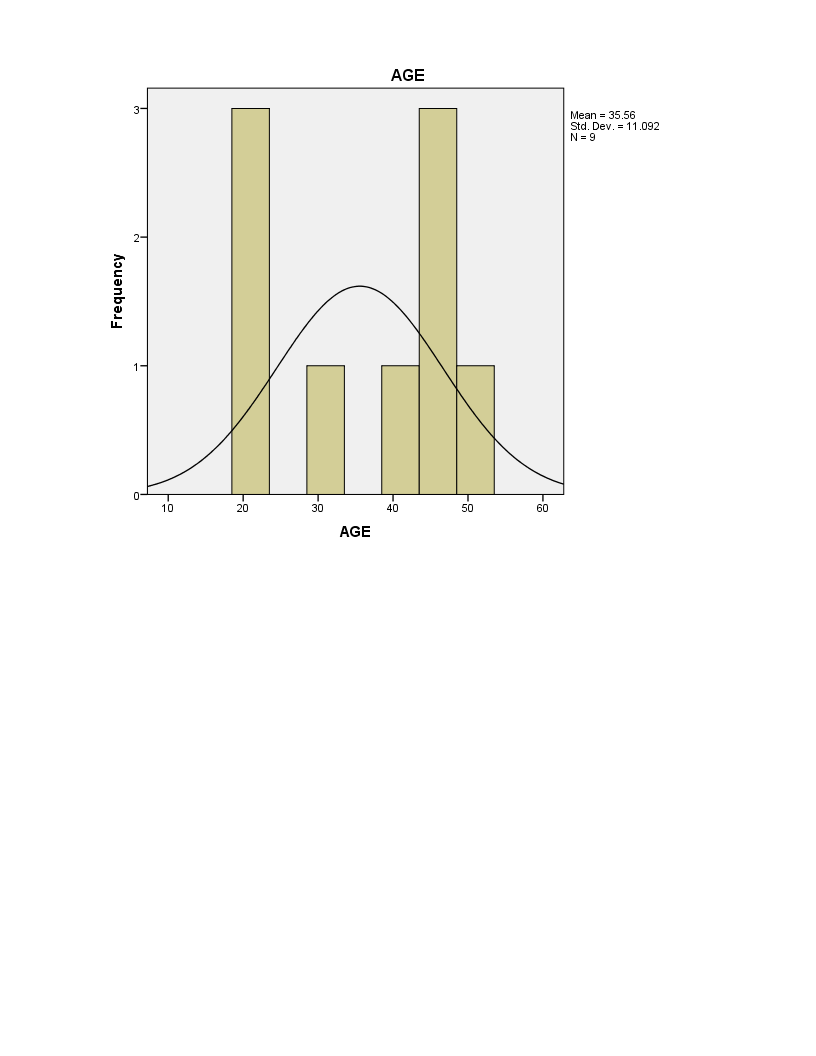

Supplement: S4 Fig — Histogram plots with normal curves applied depicting gorillas assigned a health status of “2”: a) age (years); b) interventricular septal end diastole–IVSd (cm); c) left ventricular internal diameter end diastole–LVIDd (cm); d) left ventricular posterior wall diastole–LVPWd (cm); and e) ejection fraction–EF (%). (ZIP) [file pone.0214101.s004.zip › S4A.tif]

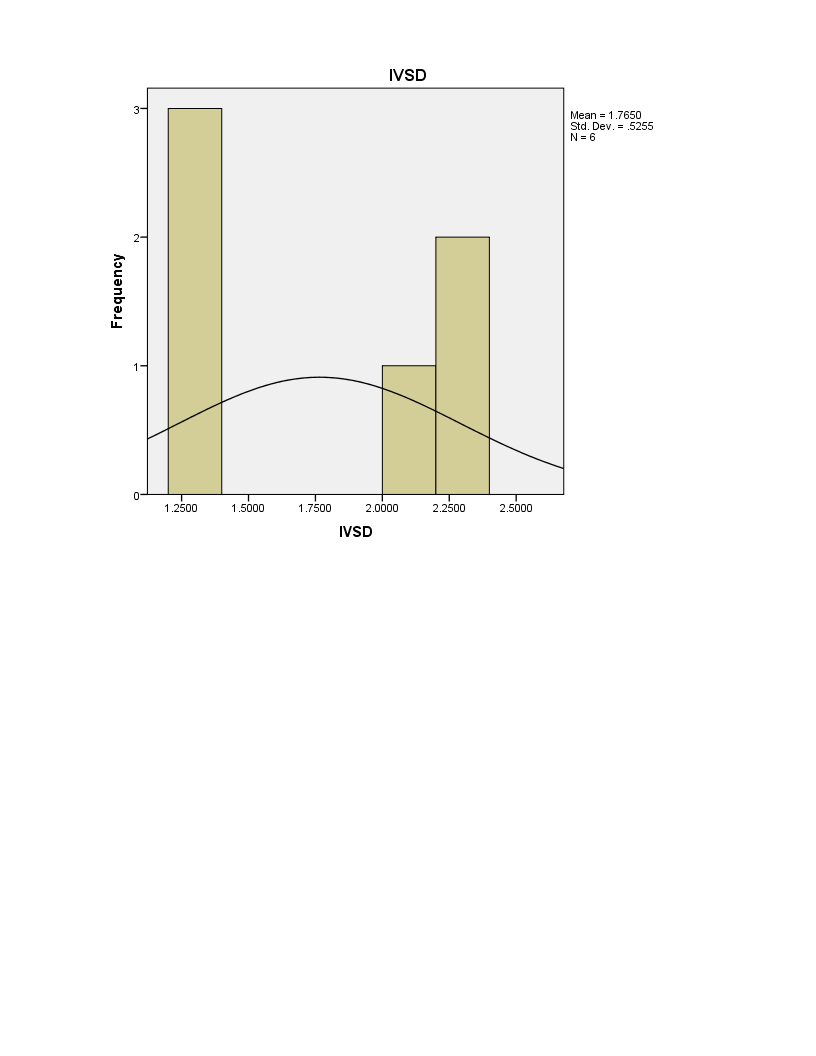

Supplement: S4 Fig — Histogram plots with normal curves applied depicting gorillas assigned a health status of “2”: a) age (years); b) interventricular septal end diastole–IVSd (cm); c) left ventricular internal diameter end diastole–LVIDd (cm); d) left ventricular posterior wall diastole–LVPWd (cm); and e) ejection fraction–EF (%). (ZIP) [file pone.0214101.s004.zip › S4B.tif]

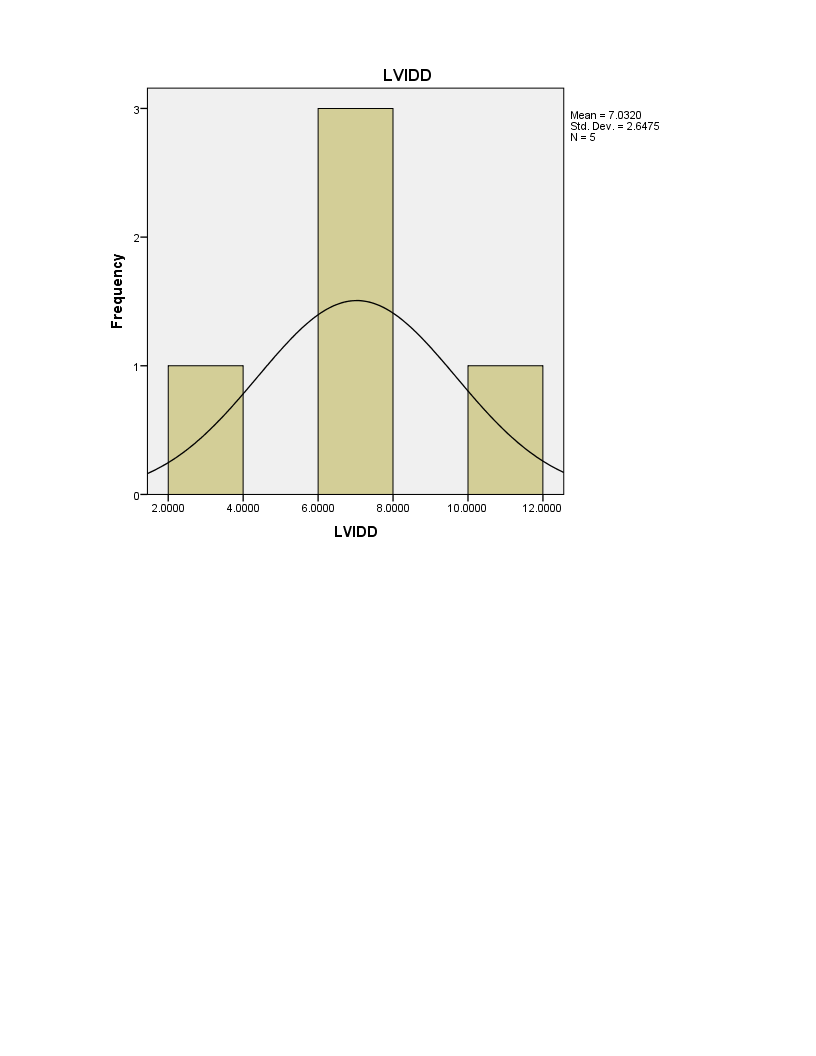

Supplement: S4 Fig — Histogram plots with normal curves applied depicting gorillas assigned a health status of “2”: a) age (years); b) interventricular septal end diastole–IVSd (cm); c) left ventricular internal diameter end diastole–LVIDd (cm); d) left ventricular posterior wall diastole–LVPWd (cm); and e) ejection fraction–EF (%). (ZIP) [file pone.0214101.s004.zip › S4C.tif]

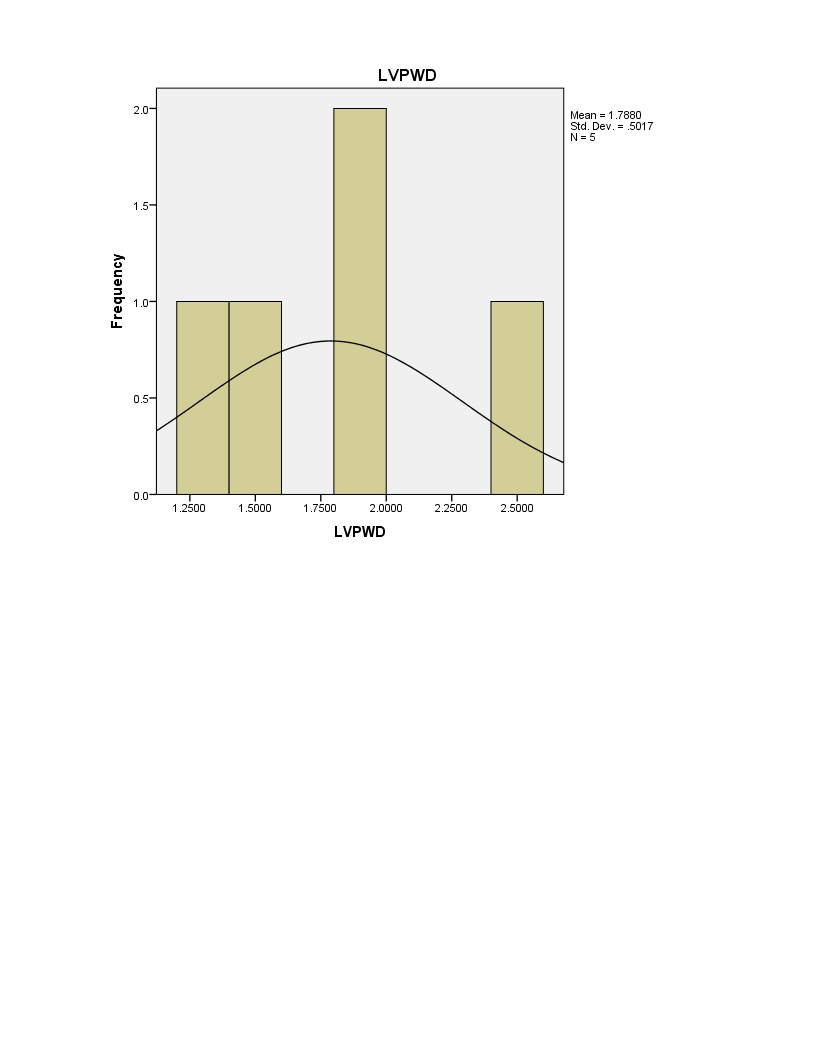

Supplement: S4 Fig — Histogram plots with normal curves applied depicting gorillas assigned a health status of “2”: a) age (years); b) interventricular septal end diastole–IVSd (cm); c) left ventricular internal diameter end diastole–LVIDd (cm); d) left ventricular posterior wall diastole–LVPWd (cm); and e) ejection fraction–EF (%). (ZIP) [file pone.0214101.s004.zip › S4D.tif]

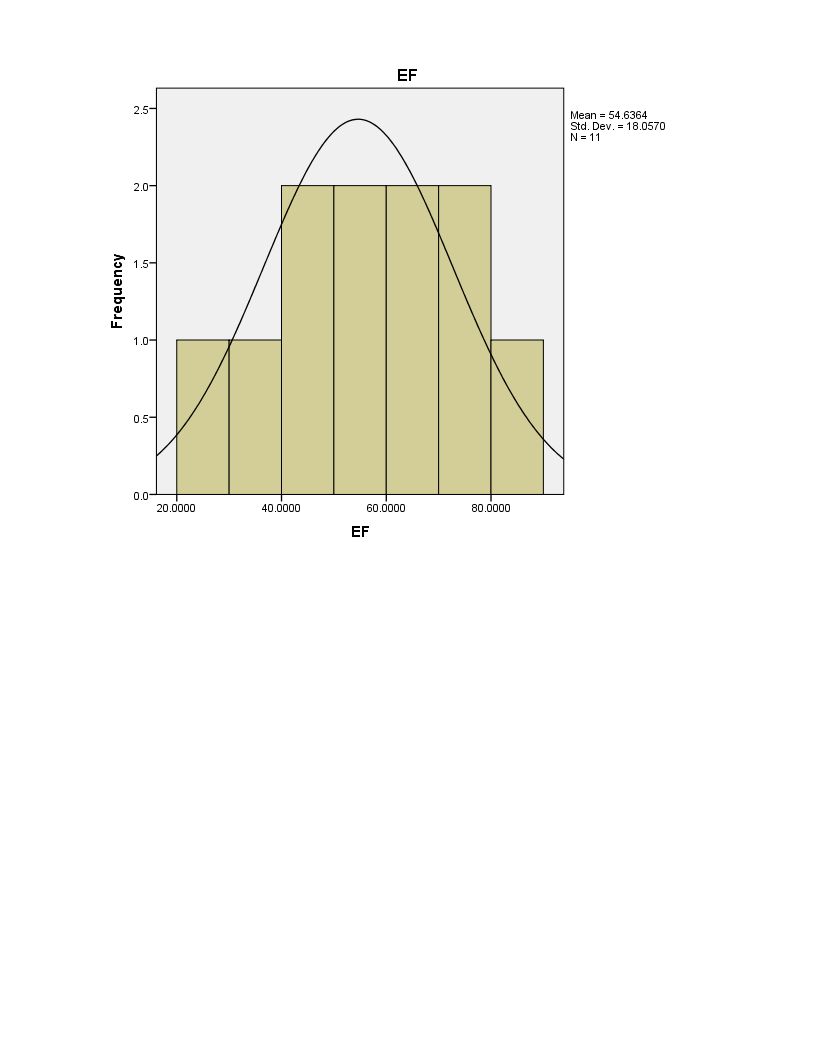

Supplement: S5 Fig — Histogram plots with normal curves applied depicting gorillas assigned a health status of “3”: a) age (years); b) interventricular septal end diastole–IVSd (cm); c) left ventricular internal diameter end diastole–LVIDd (cm); d) left ventricular posterior wall diastole–LVPWd (cm); and e) ejection fraction–EF (%). (ZIP) [file pone.0214101.s005.zip › S5E.tif]

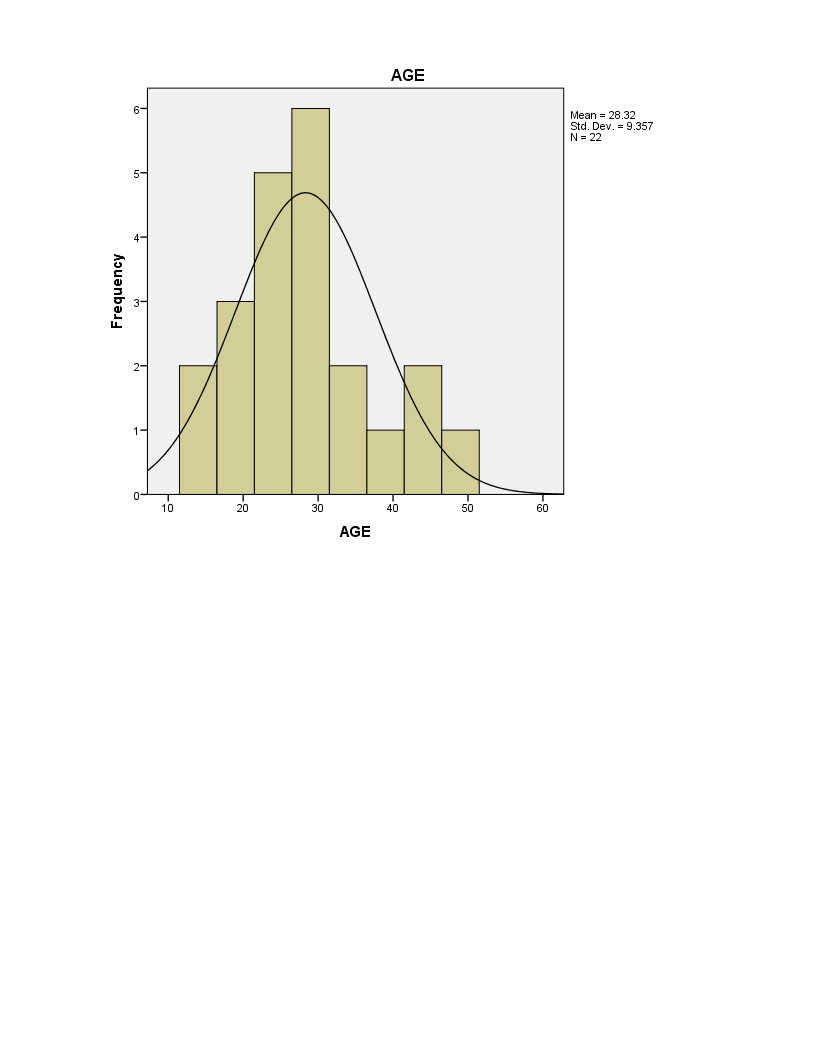

Supplement: S5 Fig — Histogram plots with normal curves applied depicting gorillas assigned a health status of “3”: a) age (years); b) interventricular septal end diastole–IVSd (cm); c) left ventricular internal diameter end diastole–LVIDd (cm); d) left ventricular posterior wall diastole–LVPWd (cm); and e) ejection fraction–EF (%). (ZIP) [file pone.0214101.s005.zip › S5A.tif]

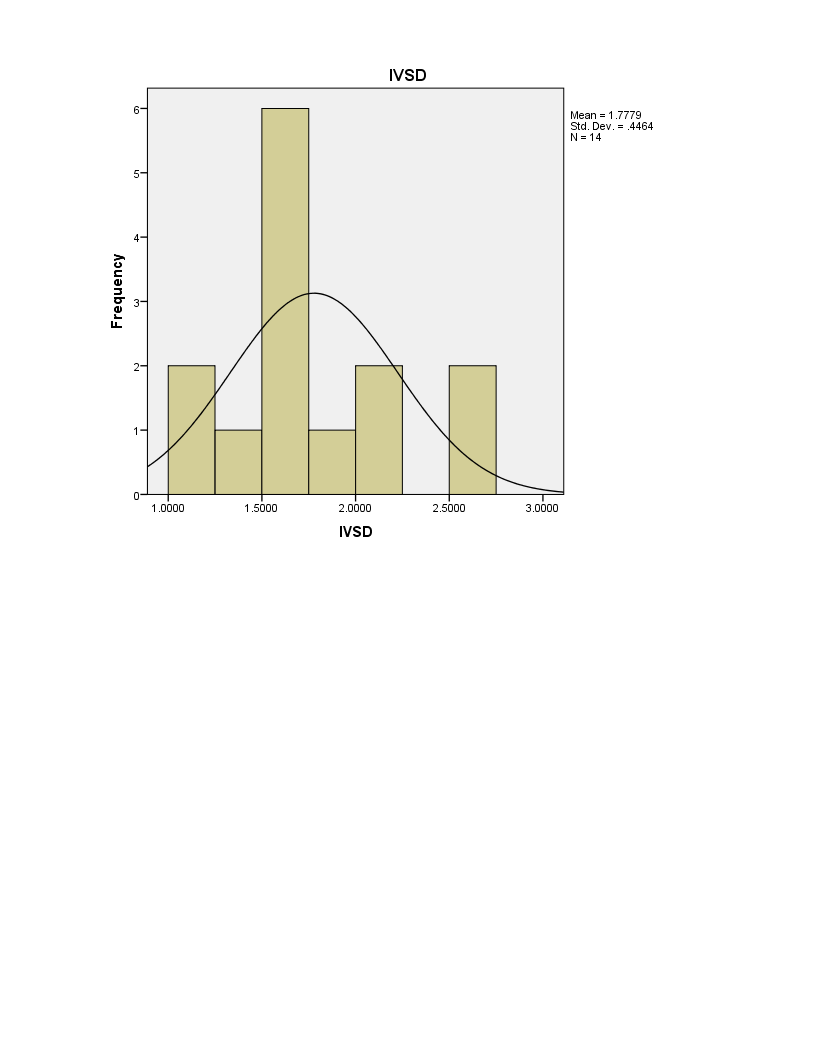

Supplement: S5 Fig — Histogram plots with normal curves applied depicting gorillas assigned a health status of “3”: a) age (years); b) interventricular septal end diastole–IVSd (cm); c) left ventricular internal diameter end diastole–LVIDd (cm); d) left ventricular posterior wall diastole–LVPWd (cm); and e) ejection fraction–EF (%). (ZIP) [file pone.0214101.s005.zip › S5B.tif]

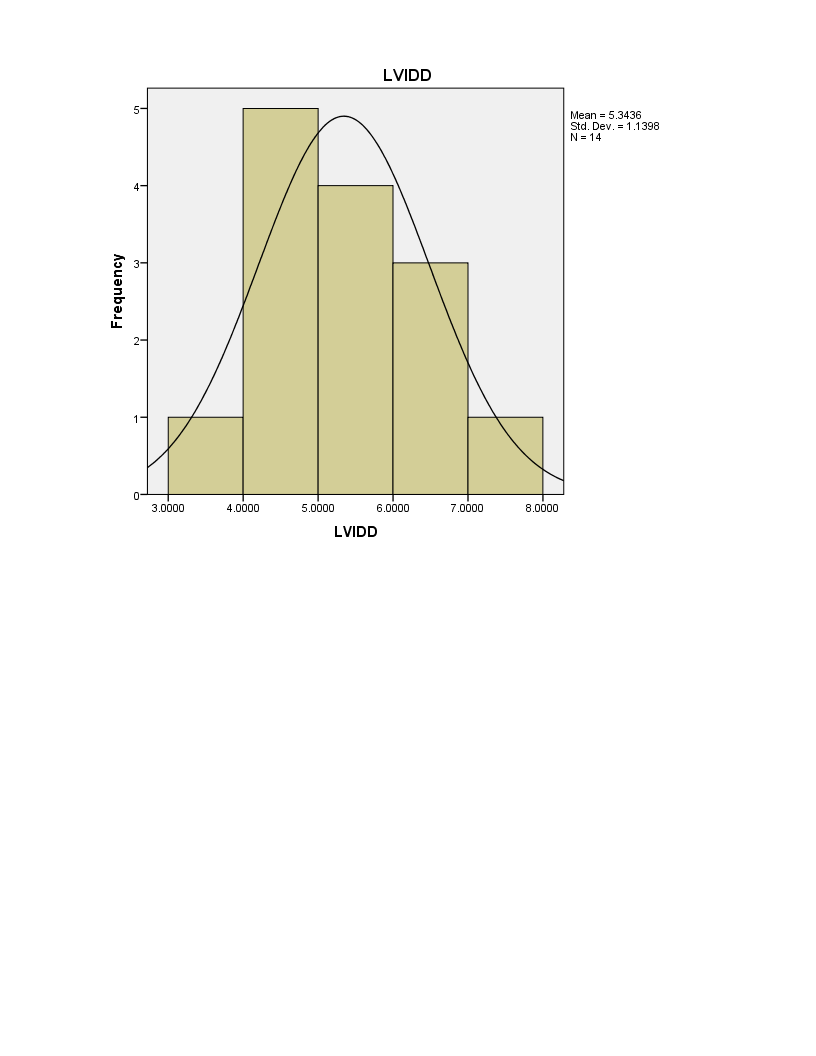

Supplement: S5 Fig — Histogram plots with normal curves applied depicting gorillas assigned a health status of “3”: a) age (years); b) interventricular septal end diastole–IVSd (cm); c) left ventricular internal diameter end diastole–LVIDd (cm); d) left ventricular posterior wall diastole–LVPWd (cm); and e) ejection fraction–EF (%). (ZIP) [file pone.0214101.s005.zip › S5C.tif]

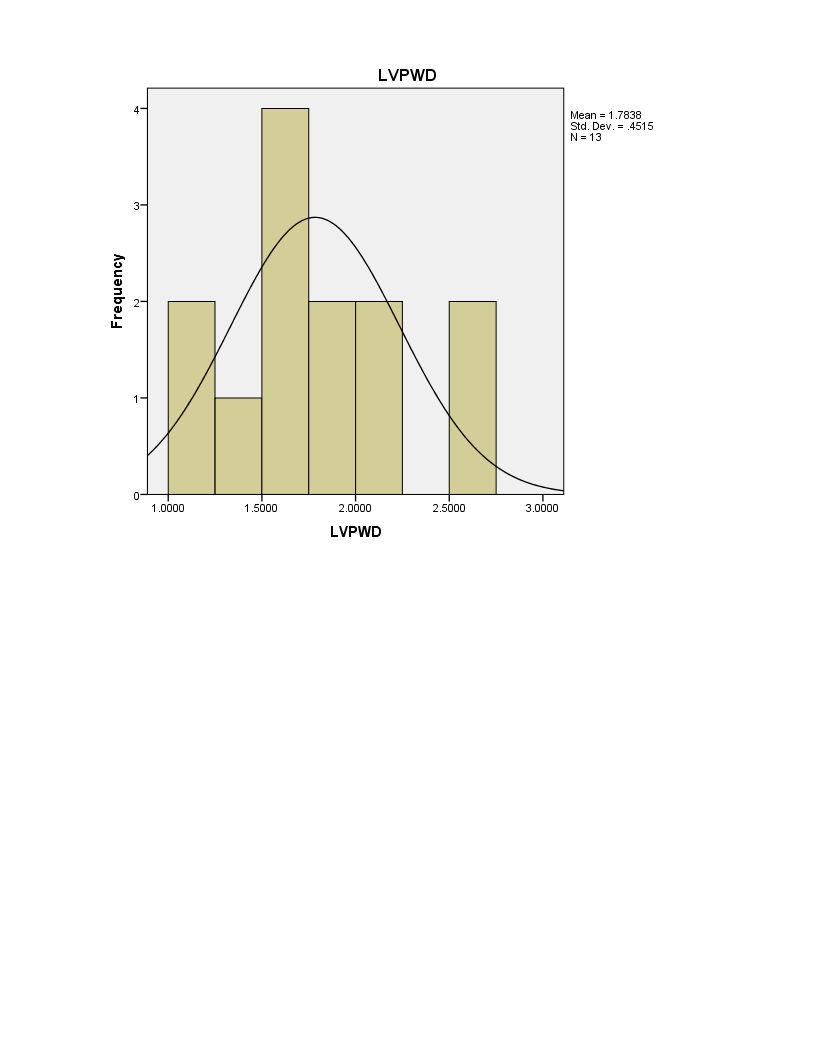

Supplement: S5 Fig — Histogram plots with normal curves applied depicting gorillas assigned a health status of “3”: a) age (years); b) interventricular septal end diastole–IVSd (cm); c) left ventricular internal diameter end diastole–LVIDd (cm); d) left ventricular posterior wall diastole–LVPWd (cm); and e) ejection fraction–EF (%). (ZIP) [file pone.0214101.s005.zip › S5D.tif]
